# Transverse Testicular Ectopy and an Incarcerated Inguinal Hernia in a 2-Month-Old Preterm Boy

**DOI:** 10.1055/s-0044-1795163

**Published:** 2024-11-28

**Authors:** Marcin Lech Kordasz, Michael Nakhleh, Christoph Matissek, Alexander Mack, Thomas Franz Krebs, Frank-Martin Haecker

**Affiliations:** 1 Department of Pediatric Surgery, Ostschweizer Kinderspital, St. Gallen, Switzerland; 2 Department of General, Visceral, Thoracic, Transplant and Pediatric Surgery, Universitätsklinikum Schleswig-Holstein, Kiel, Schleswig-Holstein, Germany; 3 Department of Pediatric Surgery, University Children's Hospital Basel, Basel, Switzerland

**Keywords:** testicular ectopy, hernia, Pediatric Surgery

## Abstract

Transverse testicular ectopy (TTE) is a rare anomaly in which both testicles descend through the same inguinal canal. Different variants of this anomaly exist, with the most common presenting as two separate spermatic cords and testicular vessel bundles. The management of this condition is challenging, as various factors have to be considered. We report on a 2-month-old preterm boy with TTE, admitted to the hospital due to an ipsilateral incarcerated inguinal hernia. Diagnostic workup included a physical examination revealing a large swelling in the right groin, ultrasound imaging that showed both testicles located in the right inguinal canal, and laboratory tests indicating a deficiency of anti-Mullerian hormone. All of these findings confirmed the diagnosis of TTE. Surgical treatment included diagnostic laparoscopy with herniorrhaphy, followed by inguinal revision with transseptal orchidopexy in a second procedure. The 12-month follow-up was uneventful. Though rare, TTE is an important differential diagnosis in case of an incarcerated hernia combined with (contralateral) empty scrotum. Pediatric surgeons must be aware of this entity. Meticulous diagnostic workup and careful surgical management are mandatory.

## Introduction

Transverse testicular ectopy (TTE) is a rare anomaly (one in four million children 1 ) in which both testicles descend through the same inguinal canal. In the majority of patients, it is an incidental finding, noticed during surgical repair of an inguinal hernia.

Other terms used in the literature to describe this condition are crossed testicular ectopy, unilateral double testis, testicular pseudoduplication, and transverse aberrant testicular maldescent. 2

Affected patients may present initially with an apparently trivial condition, such as inguinal hernia. Missing this important differential diagnosis may pose serious risks to the patient. Performing a classical inguinal hernia repair may endanger one or both testicles in case of TTE. Moreover, depending on the type of TTE, an endocrinological workup may be critical to prevent potential electrolyte imbalance.

With the advent of modern imaging tools, the condition may be diagnosed preoperatively. 3 However, the management of this disorder is not trivial, especially in case of concomitant disorders, such as incarcerated inguinal hernia. This was exactly the condition in our patient, who presented with an inguinal hernia, and diagnostic workup revealed the coexistence of TTE.

We report the diagnostic workup and surgical treatment of this 2-month-old preterm boy, who was treated for TTE and an ipsilateral incarcerated inguinal hernia.

Previous case reports describe not only incidental findings of TTE identified during repair of inguinal hernia, which were treated with extraperitoneal transposition and orchidopexy, 2 but also cases that were diagnosed preoperatively using ultrasound. 3

Since diagnosing TTE preoperatively allows to prepare the surgical management more precisely, we describe our case to raise awareness of this important differential diagnosis, which usually does not have any specific predominant typical finding.

## Case Presentation

### Patient History

Born at 33 weeks gestation, the patient developed postnatally severe respiratory distress. Due to ventilation trauma, bilateral pneumothorax developed, but the insertion of bilateral drain was effective. The further postnatal clinical course was otherwise uneventful.

### Initial Presentation

Clinical examination revealed an empty scrotum with a nonpalpable testicle on the left side, whereas a testicle could be palpated in the inguinal groin on the right side.

### Diagnostic Workup

Ultrasound imaging showed two testicle-like structures with volume of 0.2 mL, respectively, and a patent processus vaginalis on the right side. Additionally, a mild dilatation of central calyces of the right kidney was noticed. Since the child was in no distress, watchful waiting was planned to allow spontaneous descent and the patient was discharged after 1 month of hospitalization.

### First Treatment

However, 6 weeks after birth the patient presented with a huge swelling of the right groin to our emergency department.

Clinical examination confirmed the diagnosis of an incarcerated, but finally reducible, inguinal hernia on the side of TTE.

**Fig. 1:**
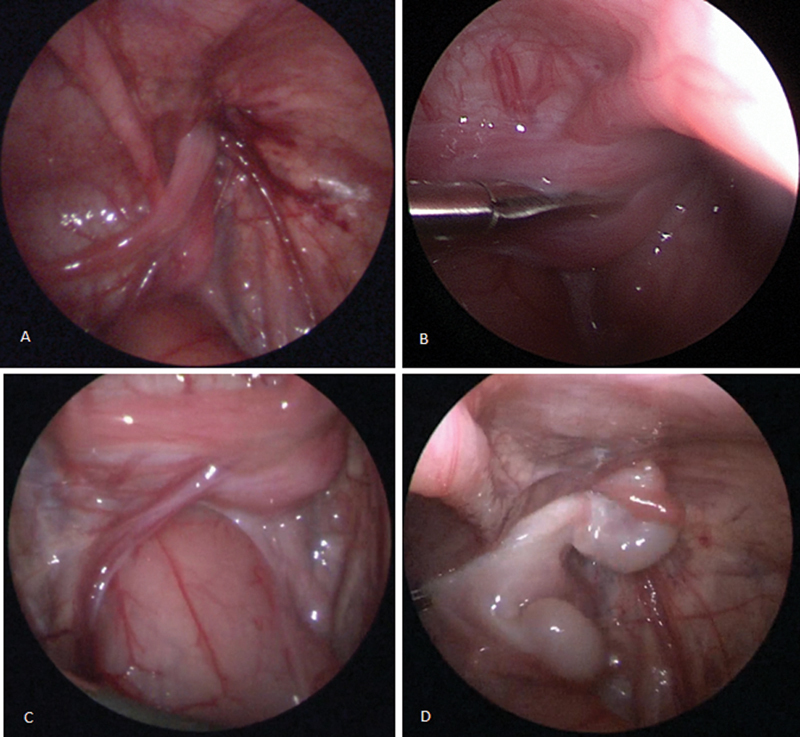
Intraoperative findings: right inguinal ring (A), paramesonephric duct remnants (B), left duct crossing retrovesical (C), and testicles luxated out of the inguinal ring (D).

Inguinal hernia repair was considered. However, closure of the hernia using an inguinal approach would definitely compromise a potential spontaneous descent of both testicles. Finally, the patient underwent diagnostic laparoscopy at the age of 43 days with a body weight of 3,500 g. During the procedure, a retrovesical structure, approximately 4 × 2 cm in size, maybe resembling a uterus, was noticed. Both testicles were luxated from the inguinal canal. They showed a volume of approximately 0.6 mL and a nearly complete testicular-epididymal dissociation ( Fig. 1 ). Moreover, both testicles were connected by a strong bridge of tissue, possibly remnants of the oviduct. The left deferent duct passed through the closed left inguinal ring and followed to the widely open right internal inguinal ring. After careful exploration, both testicles have been reinserted into the right inguinal canal and a laparoscopic percutaneous herniorrhaphy was performed.

### Recurrence

One month later, the patient presented again with a prominent inguinal swelling to our emergency department. Clinical examination confirmed diagnosis of a recurrent incarcerated inguinal hernia on the right side. However, this time, reduction was not possible. Ultrasound showed both testicles in the right inguinal canal, with volumes of 0.6 and 0.7 mL, respectively.

### Second Treatment

The patient underwent immediate inguinal herniotomy at the age of 72 days, with a body weight of 4,500 g. Both testicles originated from separate deferent ducts.

Inguinal herniotomy and bilateral transseptal orchidopexy were performed.

### Follow-Up

**Fig. 2:**
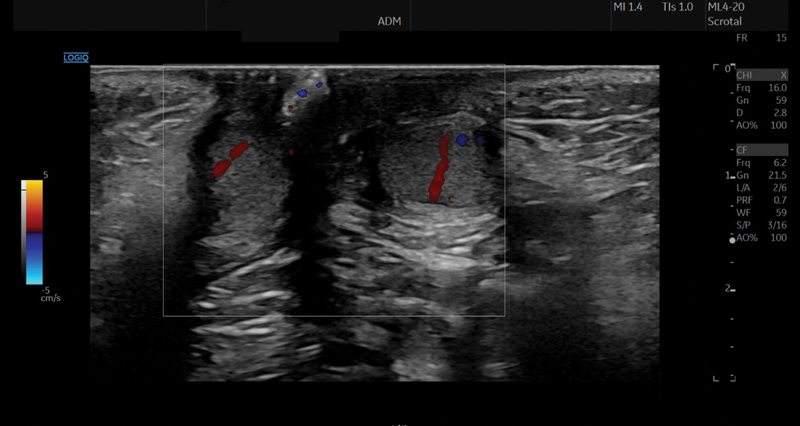
Doppler sonography 12 months postoperatively.

The postoperative course after second surgery was uneventful. Clinical examination at 6 and 12 months after surgery showed both testicles in scrotal position with symmetric volume, which was confirmed by ultrasound, including Doppler sonography of the testicles ( Fig. 2 ). The testicles grew continuously, with a volume of 0.6 mL at 6 months after surgery and 0.7 mL (right)/0.8 mL (left) at 12 months after surgery. The ultrasound performed 1 year after surgery showed no more calyceal dilation, with normal growth of both kidneys (diameter of 6.5 cm). The color Doppler sonography showed normal perfusion of both testicles.

### Genetic Analysis

Already earlier in the management, our endocrinologists and genetic specialists were involved. According to their assessment, no acute management of endocrinological conditions was necessary, but a regular workup was performed to gain insights into the pathophysiology.

Array analysis of genome revealed a normal male phenotype, without anomalies of sex differentiation. Biochemical workup revealed deficiency of anti-Mullerian hormone (AMH).

This explains the pathophysiology of TTE in our case. Together with laparoscopic findings, this allows us to classify the presented case as TTE type II.

## Discussion

TTE is a disorder with an incidence of one in four million children, 1 with both testicles descending through the same inguinal canal. It was first described in 1886 by Von Lenhossek who noticed TTE in an autopsy specimen. 4 Until now, ∼260 cases worldwide have been reported. 5

Gauderer et al have proposed a classification of TTE depending on coexisting anomalies 6 :

- Type I: Inguinal hernia only (most common, 40–50%).
- Type II: Mullerian duct structures present (30%).
- Type III: Other genitourinary anomalies, without Müllerian remnants (hypospadias, pseudohermaphroditism, and scrotal abnormalities).

Few cases of fused vas deferens have been reported. 7 In the majority of cases, separate ducts and vessels are described.

In type II TTE, which corresponds to our case, the presence of Mullerian remnants poses a mechanical resistance to physiological descent of testicles, pushing both testes into the same hemiscrotum. 8 Usually, the problem is caused by the deficiency of AMH in the Sertoli cells, which prevents degeneration of Mullerian remnants despite male phenytope. 9 Few cases are caused rather by AMH-receptor resistance. 10

It is worth noting that in most of the cases, the testicles are structurally deranged and azoospermia is commonly present. 11

This was one of the considerations why we chose watchful waiting initially. Performing a procedure on testicles that are already deranged may further compromise the long-term prognosis with a good outcome.

In TTE type II, not only low fertility poses a problem. Presence of Mullerian residues is associated with development of malignant tumors. 12

There are generally two surgical treatment options for TTE: transperitoneal and transseptal orchidopexy. The transperitoneal orchidopexy is performed by moving the ectopic testis through the root of the penis. 13 However, the length of the deferent duct is a major limitation of this technique.

The preferred technique is transseptal orchidopexy, which has been described by Bascuna et al. 14 Raj et al have proposed an algorithm of treatment of TTE: if the duct and testicular vessels are long enough, transseptal orchidopexy should be performed. If not, one should attempt to conduct an orchidopexy through the empty contralateral inguinal canal. In case of severe length mismatch, orchidopexy in the same scrotum is proposed as a solution. 15

In our case, both ducts and vessels could be easily separated and the length of the ducts was not a limitation, so a transseptal orchidopexy could be performed.

Despite the risk of malignancy developed in the remnants of Mullerian duct, the incidence of malignant transformation is too low to recommend removal of these structures. The risk of injuring very delicate blood supply to the testis while dissecting Mullerian remnants is much higher than the benefit of removal. 16 This is why we decided for closed follow-up, instead of removing the remnants during the initial surgical treatment.

Since the incidence of TTE is very low, there is a lack of universal standardized recommendations for its management. Our case confirms that transseptal orchidopexy, if possible, is a good treatment solution for TTE.

## Conclusion

TTE is a rare but complex condition. Our case of type II TTE with AMH deficiency was managed with transseptal orchidopexy, and the decision was made not to remove the paramesonephric duct remnants. The patient had an uneventful follow-up, highlighting the importance of individualized surgical planning and long-term monitoring in managing TTE cases effectively.

## Notes

Importance for the Pediatric Surgeon Transverse testicular ectopy is a rare condition that requires careful management. Transseptal orchidopexy is one of possible treatment methods, and removal of paramesonephric remnants is not necessary.
